# Influence of Milling–Electrochemical Polishing on Corrosion Resistance of NiTi Shape Memory Alloy

**DOI:** 10.3390/mi13122204

**Published:** 2022-12-12

**Authors:** Guijie Wang, Hongbin Xia, Weimin Huang, Junru Yang, Bing Liu, Liang Yuan

**Affiliations:** 1 College of Mechanical and Electronic Engineering, Shandong University of Science and Technology, Qingdao 266510, China; 2 School of Mechanical Engineering, Shandong University, Jinan 250061, China; 3 Key Laboratory of High Efficiency and Clean Mechanical Manufacture of Ministry of Education, Key National Demonstration Center for Experimental Mechanical Engineering Education, Shandong University, Jinan 250061, China; 4 College of Computer Science and Engineering, Shandong University of Science and Technology, Qingdao 266510, China

**Keywords:** NiTi shape memory alloy, milling, electrochemical polishing, corrosion resistance

## Abstract

As an important artificial implant material, the corrosion resistance of NiTi shape memory alloy is closely related to the machined surface quality. In this paper, the multiple analysis methods concerning potentiodynamic polarization, impedance spectrum and corrosion morphology are used to analyze the corrosion resistance of the alloy. The potentiodynamic polarization and impedance spectrum test results show that the conductivity and corrosion current density of electrochemical polishing surface decrease, and the polarization resistance and corrosion potential increase compared with milling. After electrochemical polishing, the surface roughness of the milling sample is decreased, and the NiTi alloy of austenite phase is transformed into TiO_2_, which improves the corrosion resistance of the alloy. In addition, there are pitting corrosion, hole corrosion and crevice corrosion morphology on the milling surface, while the pitting corrosion and hole corrosion exist on the electrochemical polishing surface. The corrosion morphology verified the analysis of potentiodynamic polarization and impedance spectrum. The multiple analysis method proposed in this paper can be used as a more accurate evaluation method for the corrosion resistance of alloy surface, avoiding the error of analysis results caused by the impedance spectrum equivalent circuit and potentiodynamic polarization following Tafel relationship.

## 1. Introduction

NiTi shape memory alloy is widely used in the medical field benefiting from excellent shape memory effect, hyperelasticity and biocompatibility [1,2,3]. However, this kind of alloy is apt to be corroded when implanted in human body, leading to the dissolution of Ni and Ti from NiTi shape memory alloy. This will produce toxins effect on human body and make the tissue around the implant appear blackening phenomenon [4]. Therefore, the satisfying corrosion resistance of the alloy is very important for the application of implants [5,6].

NiTi shape memory alloy could be corroded by the surrounding medium, and the types of corrosion morphology are divided into overall corrosion and local corrosion. The corrosion surface is mainly local corrosion, such as pitting corrosion, hole corrosion, intergranular corrosion, crevice corrosion and delamination corrosion [7,8]. The factors affecting the formation of corrosion are summarized as internal factors (surface roughness, work hardening, oxide film and stress) [9,10] and external factors (electrolytes, temperature, humidity, etc.) [11,12]. The influence of surface quality on the corrosion resistance of NiTi shape memory alloy is very complex. The corrosion resistance of NiTi shape memory alloy is closely related to surface roughness, work hardening and oxide film. Generally speaking, the corrosion potential and polarization resistance of the alloy can be obviously strengthened with the improvement of surface quality, which contributes to the corrosion resistance of NiTi shape memory alloy [13,14,15].

Hu et al. studied the corrosion behavior of NiTi shape memory alloy in 0.9% sodium chloride physiological solution by electrochemical tests [16]. They found that pitting corrosion extends to the whole surface with the increase of immersion time. Ni ions are released from the alloy surface to the solution, and the residual Ti reacts with oxygen, forming TiO_2_ in the process of corrosion. With the increase of corrosion time, the growth rate of TiO_2_ film decreases gradually, which hinders the further corrosion. The release rate of Ni ions is related to the surface structure and immersion time, and the TiO_2_ film hinders the precipitation of Ni ions. Alqarni et al. studied the electrochemical corrosion behavior of Ni52Ti48 alloys by linear polarization resistance, linear sweep voltammetry, chronoamperometry and dynamic electrochemical impedance spectroscopy in 0.9% sodium chloride solution at 37 °C [17]. The passivation layer composed of TiO_2_ has high stability, which reduces the adsorption effect of Cl ions. Furthermore, the added Co reduces the corrosion rate of NiTi shape memory alloy and improves the pitting corrosion resistance of the alloy. Xue et al. found that wear can accelerate corrosion compared with static electrochemical corrosion [18]. When the corrosion potential shifts negatively and the corrosion current density increases, the corrosion resistance of the alloy decreases. Therefore, researchers have made or coated TiO_2_ film or non-TiO_2_ film on NiTi shape memory alloy surface to further improve the corrosion resistance of the alloy through surface modification technology [19].

The effect of laser surface remelting on the corrosion resistance of the NiTi shape memory alloy was studied by Qiu et al. [20]. The dense remelting layer formed on the sur-face of the NiTi shape memory alloy after laser surface remelting. Then, the Ti/Ni and Ti^4+^/Ti ratios on the surface and corrosion potential of the alloy improved significantly, thus improving the corrosion resistance of the alloy. The laser power has a great influence on the corrosion resistance of the alloy. With the increase of laser power, the grain size and corrosion potential increase, and the corrosion current density decreases, which improves the corrosion resistance of the alloy. The surface energy of the alloy decreases from 61 mN/m to 56 mN/m, and the energy density increases from 20 J/mm^2^ to 80 J/mm^2^. The decrease of surface energy makes the corrosion potential move to a higher direction, which reduces the corrosion current density [21].

TiO_2_ nanotube film was prepared on NiTi shape memory alloy surface by electrochemical anodic oxidation [22]. The corrosion resistance of the alloy is related to the morphology, composition and microstructure of the film. The films with good nanotube structure can be prepared at 35 V, 45 °C and 10 min. After annealing at 600 °C, rutile crystals are formed, which improve the corrosion resistance of the alloy. Wang et al. prepared TiO_2_ coating containing Ca-P on NiTi shape memory alloy by plasma electrolytic oxidation in the TiO_2_ and Ca-P solution [23]. The potentiodynamic polarization curves show that, compared with the base sample, the corrosion current density of the plasma electrolytic oxidation treated sample is two orders lower. That is to say, after plasma electrolytic oxidation treatment, the TiO_2_ coating on the surface of the alloy is formed, and then the corrosion resistance of NiTi shape memory alloy is significantly improved. Fluorinated diamond-like carbon films with different CF4 contents were deposited on the polished surface of NiTi shape memory alloy by plasma immersion ion implantation deposition [24]. Fluorinated diamond-like carbon films improve the surface corrosion resistance of the NiTi shape memory alloy. With the increase of CF4 flow rate, the corrosion resistance of alloy first increases and then decreases. Ali et al. studied the corrosion behavior of NiTi shape memory alloy in 3.5% NaCl and simulated body fluid solution by carbon plasma immersion ion implantation (C-PIII) [25]. The smooth crack-free coating with a thickness of 50 nm can be formed on the surface of the alloy through C-PIII, which improves the surface stability, reduces the corrosion current density and the amount of Ni ion precipitation and improves the corrosion resistance of the alloy.

The above studies found that the diamond films, TiN films and TiO_2_ films prepared on the alloy surface by plasma immersion ion implantation, solgel and anodization can improve the corrosion resistance and reduce the release of toxic ions. However, the above method cannot effectively reduce the cutting surface roughness, so it cannot be used as the finishing process after cutting. Electrochemical polishing can reduce the cutting surface roughness and form a layer of TiO_2_ film on the surface, which contributes to the improvement of the corrosion resistance of NiTi shape memory alloy. The potentiodynamic polarization test and impedance spectroscopy test are the classical methods for analyzing electrochemical corrosion, and surface morphology is the classical method for surface analysis. In this paper, the influence of surface composition and roughness of NiTi shape memory alloy on corrosion resistance are studied by a potentiodynamic polarization test, impedance spectroscopy test and surface corrosion morphology.

## 2. Sample Preparation

**Figure 1:**
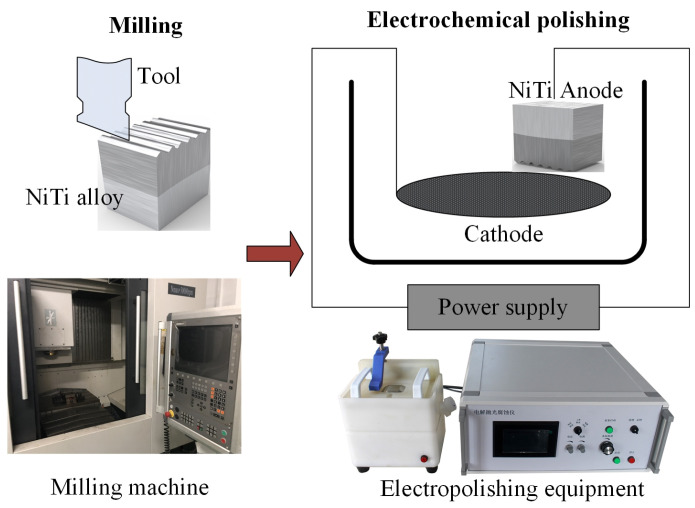
Schematic diagram of processing technology and equipment.

The selected material is composed of 69.6% austenite (B2) and 30.4% martensite (B19′) at room temperature [26]. Single factor tests were designed and carried out with vertical machining center VMC0540d (SHENYANG MACHINE TOOL Co Ltd., Shenyang, China) and electrolytic polishing equipment EDAX—TSL (Buehler, Lake Bluff, IL, USA), as shown in Figure 1 [27,28]. Cutting speed and current density are determined as the two variables considering that these two factors are always the most important process parameters for the milling process and electrochemical polishing process, respectively. A constant depth of cut (*a_p_*) = 1 mm, feed rate (*f_z_*) = 0.08 mm/r, and width of cut (*a_e_*) = 0.3 mm were selected, as well as varying cutting speed (*v_c_*) = 75, 100, 125, 150, 175, 200, 225 and 250 m/min. The electrochemical polishing process was carried out with different current density (0.5, 1, 1.5, 2, 2.5, 3, 3.5, 4.5, 5.5 and 6.5 A/cm^2^). The used electrolyte is composed of HClO_4_, CH_3_COOH, C_2_H_5_OH and C_3_H_8_O_3_ with volume ratio 82: 800: 106: 55. The electrode spacing, the polishing time and the temperature were set as 7 cm, 100 s and −20 °C, respectively.

### 2.1. Milling Process

**Figure 2:**
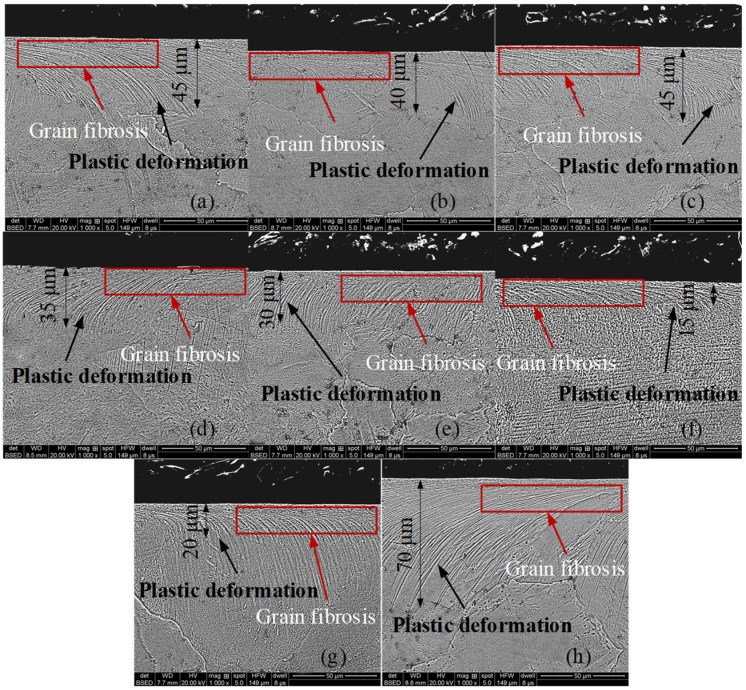
Plastic deformation and grain fibrosis of milling samples: (**a**) 75 m/min, (**b**) 100 m/min, (**c**) 125 m/min, (**d**) 150 m/min, (**e**) 175 m/min, (**f**) 200 m/min, (**g**) 225 m/min and (**h**) 250 m/min.

The influence of surface roughness and thickness of oxide film on corrosion resistance is the focus of this study, so milled samples with the least work hardening were selected to minimize the influence of work hardening on corrosion resistance. Work hardening can be evaluated by hardening degree, depth of work hardening, grain fibrosis and plastic deformation. The grain fibrosis and plastic deformation were obtained by using Nova Nano SEM450. The hardening degree, the depth of work hardening and the plastic deformation of samples processed at different cutting speeds are shown in Table 1 and Figure 2.

The positive relationship between grain fibrosis and plastic deformation with work hardening is shown in Table 1 and Figure 2. The depth of work hardening layer was increased, and the degree of work hardening was strengthened as the increase of grain fibrosis and plastic deformation.

**Figure 3:**
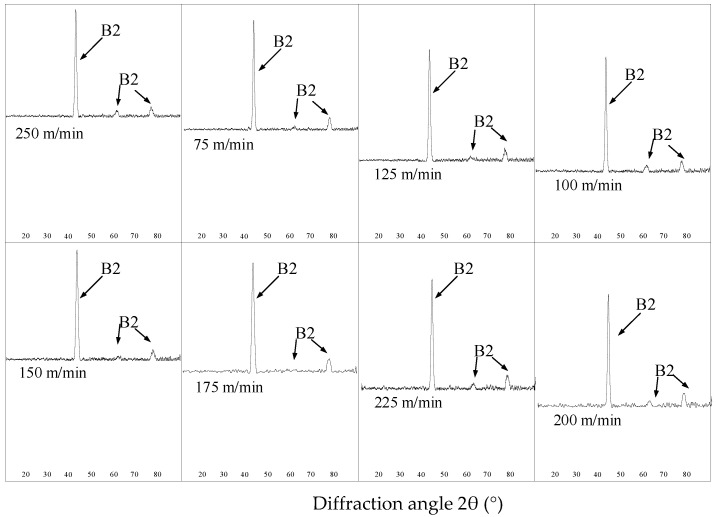
XRD pattern of the milling surface (B2: austenite).

As shown in Figure 3, the machined surfaces with different cutting speeds are composed of austenite. After the milling process, the martensite phase was transformed into the austenite phase and the hardening degree was increased.

### 2.2. Electrochemical Polishing

**Figure 4:**
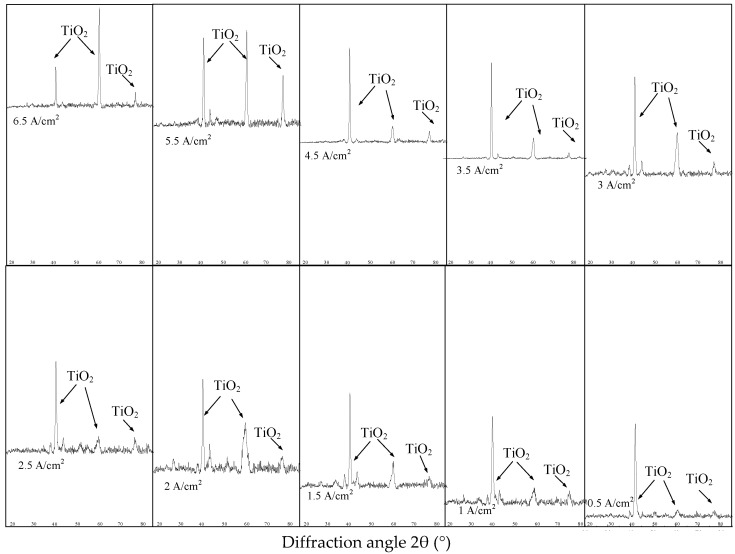
XRD patterns of electrochemical polishing surface.

As shown in Table 1 and Figure 2, the samples milled with 200 m/min cutting speed show the least obvious surface work hardening and the smallest depth of hardening layer and plastic deformation layer. Then, samples milled with 200 m/min cutting speed were selected for electrochemical polishing. As shown in Figure 4, after electrochemical polishing with different current density, the austenite phase (B2) in the milled surface of NiTi alloy disappeared, and the oxide TiO_2_ was generated.

**Figure 5:**
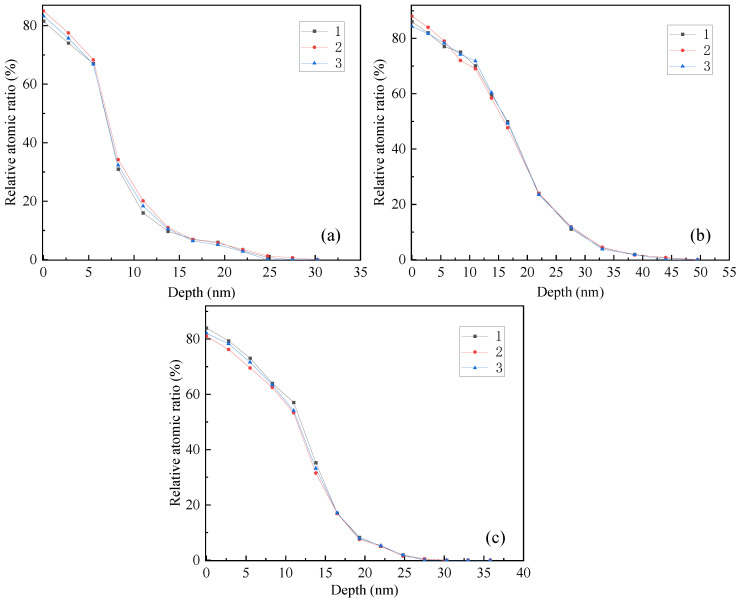
Distribution of oxygen on NiTi alloy surface along depth direction: (**a**) 1 A/cm^2^, (**b**) 3 A/cm^2^ and (**c**) 6.5 A/cm^2^.

After the polishing process, Ni was changed into Ni^2+^ and entered into the solution; Ti was changed into Ti^4+^, and an oxide film TiO_2_ was formed on the milled surface, which can affect the corrosion resistance of the alloy. The oxygen is detected by PHI680 auger nanoprobe scanning (The probe area is 100 μm, and the sputter depth reference is the known thickness SiO_2_/Si wafer), and its distribution along the depth direction is measured three times, as shown in Figure 5. The average thickness of oxide film is shown in Table 2.

**Figure 6:**
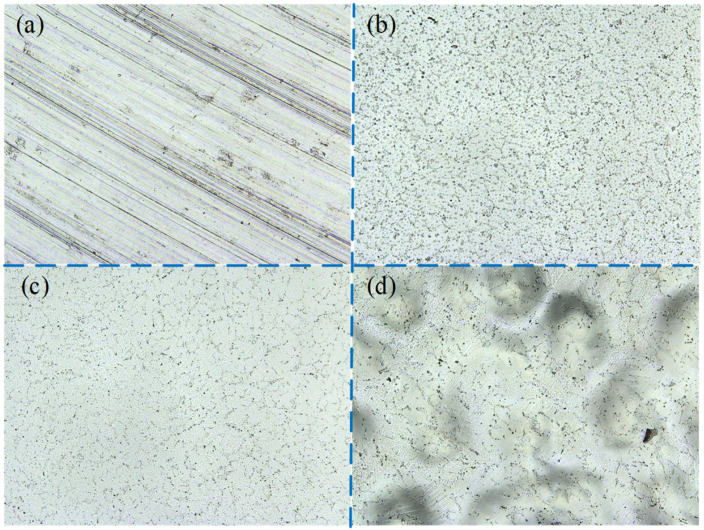
Surface morphology of electrochemical polishing: (**a**) 200 m/min milled specimen, (**b**) 1 A/cm^2^, (**c**) 3 A/cm^2^ and (**d**) 6.5 A/cm^2^.

With the increase of current density, the thickness of oxide film first increases and then decreases. As shown in Figure 6b, the alloy shows active dissolution state when the current density is 1 A/cm^2^. The dissolution rate of NiTi alloy (as anode) is less than the diffusion rate of metal ions in the solution, and there is no stable viscous liquid film on the alloy surface, so the thickness of the oxide film is relatively thin. When the current density is 3 A/cm^2^, a stable viscous liquid film layer is formed on the surface of the alloy with the dissolution of the alloy, and the surface tends to be smooth, as shown in Figure 6c. At this time, a dense oxide film composed of TiO_2_ was formed on the surface with a thickness of 17.3 nm.

However, when the current density further increases to 6.5 A/cm^2^, the polishing mechanism has pitting and becomes a viscous liquid film. At this time, the anodic reaction is violent, the oxygen precipitation rate increases, the relatively stable viscous liquid film layer is broken, the pits shown in Figure 6d are formed on the alloy surface and the oxide film thickness decreases.

### 2.3. Sample Selection and Test Parameters

Four kinds of samples (M1 is milled sample; P1, P2 and P3 are electrochemical polished samples) were selected to carry out corrosion tests. The process parameters, the oxide film thickness and the surface roughness of test samples are shown in Table 3. Surface roughness was measured by using white light interferometer Veeco NT9300 (Veeco Instruments Inc, Plainview, NY, USA), and the final surface roughness was obtained by averaging three measurements on different areas.

**Figure 7:**
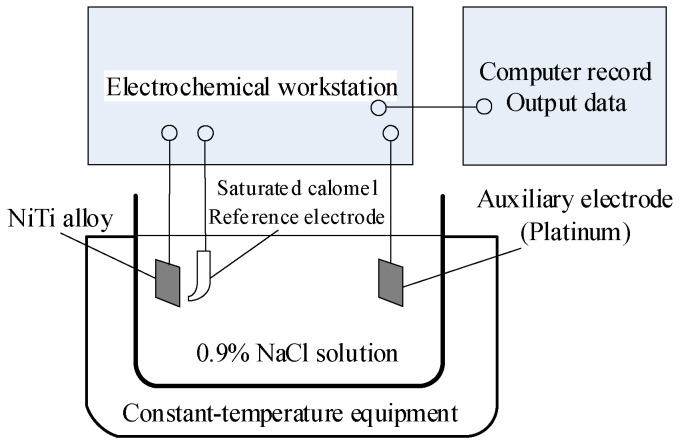
Schematic diagram of electrochemical corrosion test device.

The corrosion behavior of the samples is characterized by corrosion potential (*E_corr_*), corrosion current density (*i_corr_*) and polarization resistance (*R_p_*) to clarify the influence mechanism of the milling–electrochemical polishing process on the corrosion resistance of NiTi shape memory alloy. NiTi alloy was chosen as the working electrode, saturated calomel electrode (SCE) was chosen as the reference electrode and platinum plate was set as the auxiliary electrode, as shown in Figure 7. The NiTi alloy is exposed to 0.9% NaCl solution at 38 °C to reveal the effect of process technology on the corrosion resistance of the alloy, analyze the surface corrosion morphology of the alloy and then verify the theoretical calculation results of potentiodynamic polarization and impedance spectroscopy. The working electrode is immersed in the electrolyte for 45 min to stabilize the open circuit potential (OCP). The scanning range of potential, scanning speed and bath temperature are shown in Table 4.

## 3. Results and Discussions

### 3.1. Potentiodynamic Polarization Analysis of NiTi Alloy Surface

**Figure 8:**
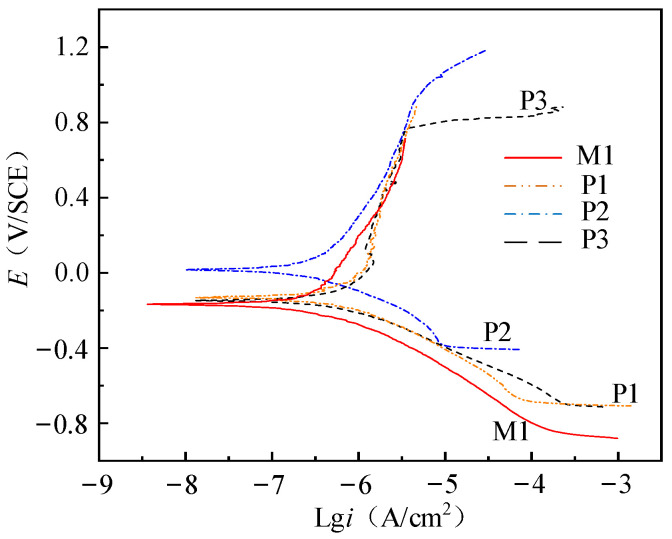
Potentiodynamic polarization curves of milling and electrochemical polishing samples.

As shown in Figure 8, the potentiodynamic polarization curves of samples (M1, P1, P2 and P3) in 0.9% sodium chloride solution are obtained.

Initially, with the process of constant rate increase of potential, the sample exists as a cathode. The potential corresponding to the intersection of anodic polarization curve and cathodic polarization curve in Figure 8 is the corrosion potential (*E_corr_*). When the potential exceeds the *E_corr_*, the sample exists as an anode and corrosion occurs.

The polarization resistance *R_p_* is obtained from the slope of polarization curve in Figure 8, as shown in Equation (1).

(1) $$R_{p}=\frac{\Delta E}{\Delta i}$$

The anodic current density (*i_a_*) and cathodic current density (*i_c_*) are expressed by Equations (2) and (3), respectively, when polarization starts from corrosion potential.

(2) $$i_{a}=i_{corr}\exp\left[\frac{2.303\left(E-E_{corr}\right)}{\beta_{a}}\right]$$

(3) $$i_{c}=i_{corr}\exp\left[\frac{-2.303\left(E-E_{corr}\right)}{\beta_{c}}\right]$$

where *i_corr_* is the corrosion current density of the sample in the electrolyte (A/cm^2^), and *β_a_* and *β_c_* are the anode polarization constant and cathode polarization constant (mV), respectively.

The kinetic expression of electrochemical corrosion (Butler–Volmer equation) is shown in Equation (4) when the applied current density is *i* = *i_a_* − *i_c_*.

(4) $$i=i_{corr}\left\{\exp\left[\frac{2.303\left(E-E_{corr}\right)}{\beta_{a}}\right]\right.-\left.\exp\left[\frac{-2.303\left(E-E_{corr}\right)}{\beta_{c}}\right]\right\}$$

The polarization resistance of Equation (1) is taken as the reciprocal, and Equation (5) is obtained by differentiating *E*.

(5) $$\frac{di}{dE}=2.303i_{corr}\left|{}_{-\beta_{a}{}^{-1}\exp\left[2.303\left(E-E_{corr}\right)/\beta_{c}\right]}^{\beta_{a}{}^{-1}\exp\left[2.303\left(E-E_{corr}\right)/\beta_{a}\right]}\right.$$

Therefore, the polarization resistance *R_p_* is calculated from Equations (6) and (7) when *E* = *E_corr_*.

(6) $${\left(\frac{di}{dE}\right)}_{E=Ecorr}=2.303i_{corr}\left(\frac{\beta_{a}+\beta_{c}}{\beta_{a}\beta_{c}}\right)$$

(7) $$R_{p}={\left(\frac{di}{dE}\right)}_{E_{=Ecorr}}^{-1}=\frac{\beta_{a}\beta_{c}}{2.303\left(\beta_{a}+\beta_{c}\right)i_{corr}}$$

where *R_p_* is the polarization resistance (Ω·cm^2^).

The corrosion parameters as shown in Table 5 are obtained by Tafel extrapolation and least square method: corrosion potential (*E_corr_*), corrosion current density (*i_corr_*), anodic polarization constant (*β_a_*) and cathodic polarization constant (*β_c_*). The polarization resistance (*R_p_*) is calculated by Equation (7).

**Figure 9:**
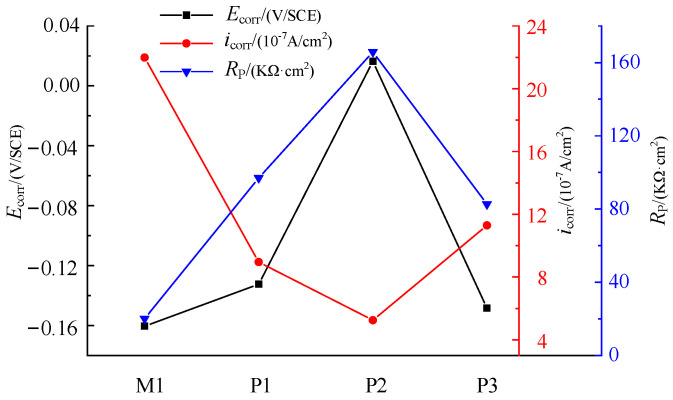
Variation of corrosion parameters of milling and electrochemical polishing samples.

The results show that the corrosion resistance of NiTi alloy electrochemical polished sample is better than that of milled samples, and the corrosion resistance of the electrochemical polished sample P2 with current density of 3 A/cm^2^ is better than other samples. As shown in Figure 9, the corrosion potential and polarization resistance of M1, P1 and P2 gradually increase, and the corrosion current density gradually decreases, indicating that the process parameters have a great influence on the corrosion resistance of the samples. As shown in Table 5, compared with sample M1, the surface roughness of P1 and P2 decrease to 0.501 μm and 0.217 μm, respectively, and the thickness of oxide film of P1 and P2 increase to 7.1 nm and 17.3 nm, respectively. The passivation layer composed of TiO_2_ has high stability, and the smaller surface roughness can reduce the adsorption effect of Cl ions. After electrochemical polishing, the decrease of surface roughness and the increase of oxide film thickness can significantly improve the corrosion resistance of the sample.

The corrosion potential and polarization resistance of P3 decrease, and the corrosion current density increases compared with P2, which makes the sample more prone to corrosion. As the current density of electrochemical polishing increases, the polishing machine changes from the action of viscous liquid film to the joint action of pitting corrosion and viscous liquid film. The thickness of surface oxide film decreases, and the surface roughness increases significantly, which further reduces the corrosion resistance of the sample. Therefore, the selection of appropriate current density is very important for the corrosion resistance of NiTi alloy.

When the surface roughness increases, the corrosive substance is easier to deposit on the surface, and the contact area between the surface and the corrosive liquid increases, which reduces the corrosion resistance of the sample. With the increase of oxide film thickness, the contact between electrolyte and metal internal materials is hindered, which increases the corrosion resistance of the sample.

The corrosion potential of P3 is close to that of M1, but the polarization resistance of P3 is 4.1 times that of M1, and the corrosion current density of M1 is 1.95 times that of P3, which means that M1 is more prone to corrosion. Therefore, even if the current density of electrochemical polishing is too high and there are pits on the surface, the corrosion resistance of the sample is obviously better than that of the milled sample. In order to ensure the accuracy of the result analysis, polarization resistance is selected as the parameter to evaluate the corrosion performance.

### 3.2. Impedance Spectrum Analysis of NiTi Alloy Surface

Electrochemical impedance can reflect the surface corrosion behavior according to the corrosion process and circuit simulation analysis of AC impedance data. As shown in Equations (8) and (9), *Z*(*w*) is the transfer function between the excitation potential and response current, which reflects the impedance of the reaction system.

(8) $$Z\left(w\right)=\frac{E(t)}{I(t)}=Z\prime \left(w\right)+jZ\prime \prime \left(w\right)$$

(9) $$\left|Z\left(w\right)\right|=\sqrt{{\left[Z\prime \left(w\right)\right]}^{2}+{\left[Z\prime \prime \left(w\right)\right]}^{2}}$$

where *w* (*w* = 2*πf*) is the angular frequency (Hz), *E*(*t*) is the potential (V), *I* is the current (A), *t* is the time (s), *Z′* is the true part (kΩ·cm^2^) and *Z″* is the imaginary part (kΩ·cm^2^).

**Figure 10:**
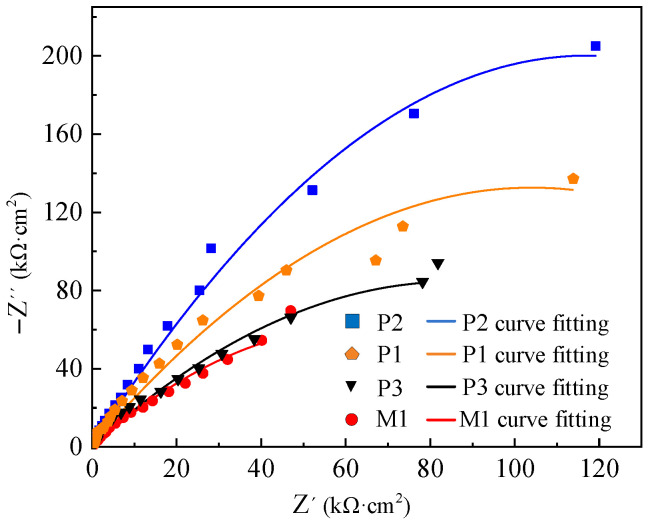
Impedance test and Nyquist spectrum of milling and electrochemical polishing samples.

**Figure 11:**
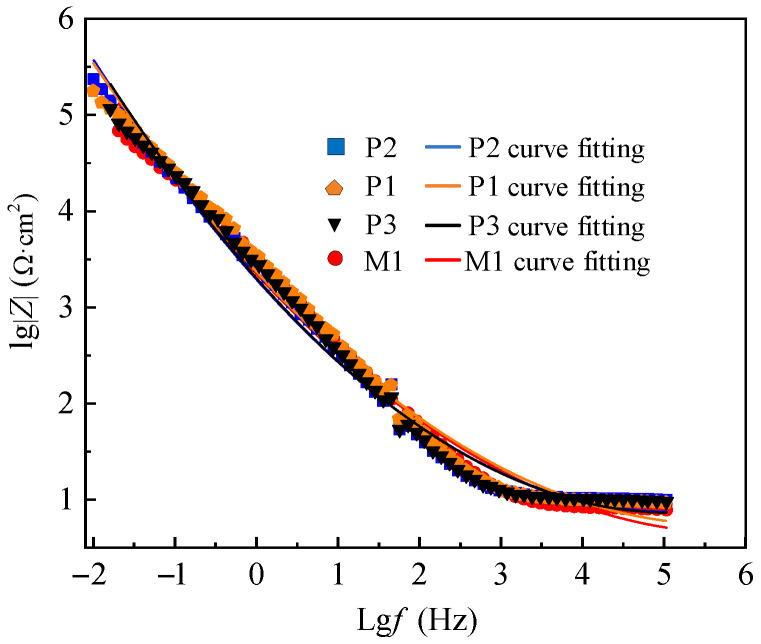
Impedance test and Bode spectrum of milling and electrochemical polishing samples.

The electrochemical impedance test results of M1, P1, P2 and P3 are presented by Nyquist and Bode patterns, as shown in Figure 10 and Figure 11, respectively.

The size of the impedance semicircle in Figure 10 reflects the corrosion resistance of the specimen (the larger the impedance semicircle is, the better the corrosion resistance of the specimen will be). The impedance semicircle is different between M and P samples, which is related to the oxide film thickness and surface roughness. The results show that the impedance semicircle of sample P is larger than that of sample M, so the corrosion resistance of sample P is better than that of sample M. The surface roughness of electrochemical polished samples decreases, and the thickness of the oxide film increases, which enhances the corrosion resistance of the sample. However, the impedance semicircle of P3 is close to that of M1, which indicates that the corrosion resistance of P3 and M1 is similar. The reason is that there are corrosion pits on the surface of the sample when the current density is too high, which reduces the corrosion resistance of the sample. The impedance semicircle of electrochemical polishing sample P2 is obviously larger than that of other electrochemical polishing samples. That is, when the current density of 3 A/cm^2^, electrode spacing of 7 cm, polishing time of 100 s and temperature of −20 °C are selected for electrochemical polishing, the corrosion resistance of the sample is relatively better.

The difference of corrosion resistance of M1, P1, P2 and P3 is also shown in Bode spectrum of Figure 11. The size of *lg*|*Z*| in the figure reflects the corrosion resistance of the sample, and the corrosion resistance of P2, P1, P3 and M1 decreases in turn.

**Figure 12:**
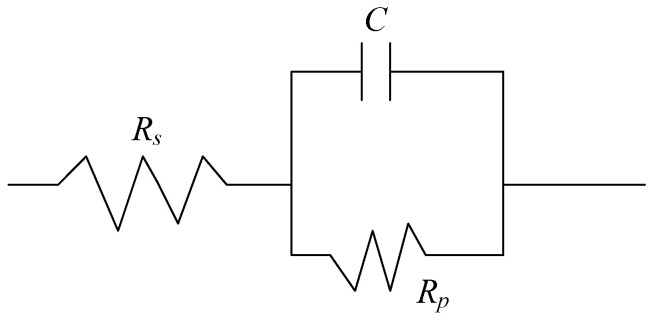
Equivalent circuit model of milling and electrochemical polishing samples.

As shown in Figure 12, the equivalent circuit model of the sample in the electrolyte was established according to the Nyquist and Bode spectra. Therefore, the impedance *Z*(*w*) of the reaction system can be calculated from Equation (10).

(10) $$Z\left(w\right)=\left(R_{s}+\frac{R_{p}}{1-w^{2}C^{2}R_{p}^{2}}\right)-j\left(\frac{wCR_{p}^{2}}{1-w^{2}C^{2}R_{p}^{2}}\right)$$

where *R_s_* is the solution resistance (Ω·cm^2^); *C* is the interface capacitance of electrode surface, hindering the flow of current (F/cm^2^); and *R_p_* is the polarization resistance (Ω·cm^2^).

*Z*(*w*_0_) and *Z*(*w*_−_) are calculated by Equation (11) and Equation (12), respectively, in the low-frequency stage (*w* = 0) and high-frequency stage (*w* = ∞).

(11) $$Z\left(w_{0}\right)=R_{s}+R_{p}(w=0)$$

(12) $$Z\left(w_{\infty }\right)=R_{s}(w=\infty )$$

Therefore, the polarization resistance *R_p_* can be calculated by Equation (13).

(13) $$R_{p}=Z\left(w_{0}\right)-R_{s}=Z\left(w_{0}\right)-Z\left(w_{\infty }\right)$$

The curve fitting results of the equivalent circuit model are shown in Figure 10 and Figure 11, which coincide well with the measured data. The solution impedance *R_s_* and polarization resistance *R_p_* of the sample in Table 6 are obtained according to Bode spectrum in Figure 12 and Equations (11) and (12).

**Figure 13:**
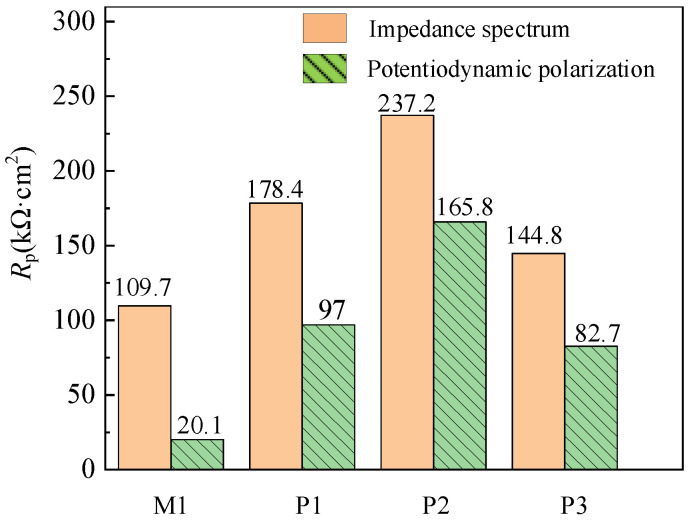
Polarization resistance of milling and electrochemical polishing samples.

As shown in Figure 13, the polarization resistance *R_p_* calculated theoretically by establishing equivalent circuit model in impedance test is significantly different from that obtained by Tafel extrapolation and least square method in the dynamic potential polarization. It is assumed that the reaction rate of anode and cathode in the process of corrosion follows the Tafel formula in the potentiodynamic polarization test, but in fact most of the corrosion systems do not meet this condition, so the polarization resistance measured by impedance spectrum is different from that measured by potentiodynamic polarization.

The difference between M1, P1, P2 and P3 of samples is 89.6, 81.4, 71.4 and 62.1 kΩ·cm^2^, respectively. However, the change trend of corrosion resistance is the same, and the polarization resistance of P2, P1, P3 and M1 decreases in turn. Therefore, it is accurate to select polarization resistance as the corrosion performance evaluation parameter of samples with different processing technologies.

### 3.3. Corrosion Morphology Analysis of NiTi Alloy Surface

**Figure 14:**
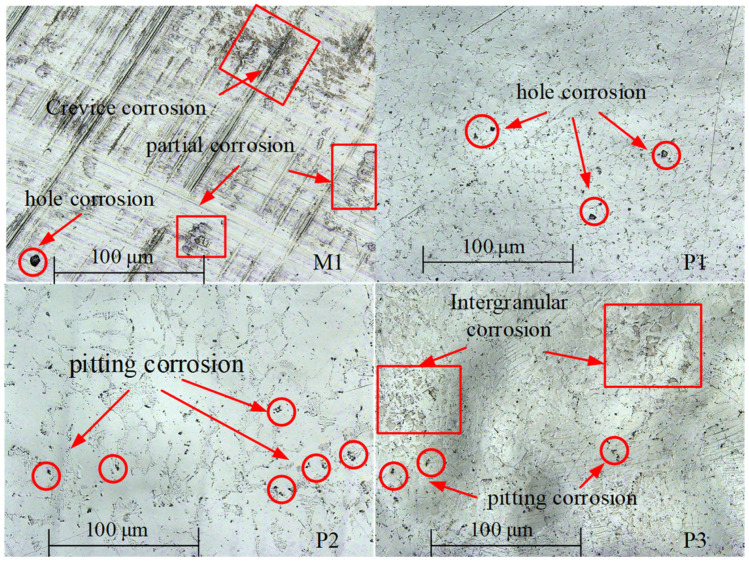
Corrosion morphology of milling and electrochemical polishing samples.

The surface corrosion morphology of NiTi alloy was analyzed to reveal the influence of processing on the corrosion resistance, and the potentiodynamic polarization and impedance spectroscopy were verified. The surface corrosion morphology of the samples is different, as shown in Figure 14.

There are pitting, hole and crevice corrosion on the surface of M1; pitting and hole corrosion on the surface of P1; pitting corrosion on the surface of P2; and pitting and intergranular corrosion on the surface of P3. The oxide film on the surface of milled sample M1 is thin and easy to be damaged, resulting in pitting corrosion and hole corrosion. The surface roughness of sample M1 is large, and the groove caused by milling process is obvious, which makes the corrosive liquid easy to gather in the depression. In this case, the corrosion of the sample is accelerated, and crevice corrosion morphology is formed. Therefore, the corrosion resistance of milled samples is poor. After electrochemical polishing, the oxide film on the surface of sample P1 is thinner than that of sample P2, and pitting and hole corrosion is easy to occur.

The thickness of the oxide film of P3 is larger than that of P1, which increases the corrosion resistance of the sample. However, the test results show that the corrosion resistance of P1 is better than that of P3. The reason is that the polishing mechanism changes to the interaction of viscous liquid film and pitting when the current density further increases to 6.5 A/cm^2^, and there are many pits on P3 surface.

The results of potentiodynamic polarization and impedance spectrum were verified by the surface corrosion morphology of the NiTi alloy. The corrosion resistance of electrochemically polished surface is significantly higher than that of milling surface. However, when the current density is too high, intergranular corrosion is easy to occur on the surface of the NiTi alloy, and the bonding force between grains is reduced, thus reducing the corrosion resistance of the NiTi alloy. Therefore, it is very important to select appropriate process parameters for corrosion of the alloy surface.

## 4. Conclusions

The effect of the milling–electrochemical polishing process on corrosion resistance of NiTi alloy was studied based on the multiple analysis of potentiodynamic polarization, impedance spectrum and corrosion morphology in 0.9% NaCl solution at 38 °C. The main findings in this paper can be summarized as following:

(1) The surface roughness with 200 m/min cutting speed, 1 mm axial depth of cut, 0.3 mm radial depth of cut, 0.08 mm/r feed rate, 7 cm electrode spacing, 100 s polishing time and 3 A/cm^2^ current density (the electrolyte volume ratio is HClO_4_: CH_3_COOH: C_2_H_5_OH: C_3_H_8_O_3_ = 82: 800: 106: 55, at −20 °C temperature) is as low as 0.217 μm, and the thickness of TiO_2_ film reaches 17.3 nm.
(2) After electrochemical polishing, the surface roughness decreases and TiO_2_ film is formed, which can not only reduce the conductivity and corrosion current density, but also increase the polarization resistance and corrosion potential, thereby improving the corrosion resistance of NiTi alloy surface.
(3) The corrosion morphology of the milled surface is different from that of the electrochemical polished surface. There are pitting corrosion, hole corrosion and crevice corrosion morphology on the milled surface, while only pitting corrosion, hole corrosion and intergranular corrosion exist on the electrochemical polished surface, which has a smaller corrosion area. Therefore, electrochemical polished surfaces have relatively better corrosion resistance than milled surfaces.
(4) The multiple analysis method proposed in this paper provides a more accurate evaluation method for the corrosion resistance of alloy surface, avoiding the error of analysis results caused by the impedance spectrum equivalent circuit and potentiodynamic polarization following the Tafel relationship.

## Figures and Tables

**Table 1:** Depth of work hardening layer (*D_H_*) and hardening degree (*N*) of milled surface.

*v_c_* (m/min)	*D_H_* (μm)				*N*			
	1	2	3	Average	1	2	3	Average
75	455	435	460	450	115.5	122.5	113.0	117.0
100	370	380	370	373	111.5	108.5	99.5	106.5
125	425	435	420	427	110.5	109.5	119.0	113.0
150	325	330	320	325	98.5	101.5	94.0	98.0
175	275	280	270	275	87.5	90.5	87.5	88.5
200	145	140	150	145	76.5	77.0	80.5	78.0
225	195	205	205	202	83.5	87.0	81.5	84.0
250	525	535	525	528	124.5	125.5	120.5	123.5

The number 1–3 represents different measurements.

**Table 2:** The thickness of oxide film on the electrochemical polishing surface.

Current Density (A/cm^2^)	Oxide Film Thickness (nm)
1	7.1
3	17.3
6.5	12.0

**Table 3:** Oxide film thickness and surface roughness of test samples.

Sample	Process Parameters	Oxide Film Thickness (nm)	Surface Roughness (μm)
M1	200 m/min	3.7	0.598
P1	1 A/cm^2^	7.1	0.501
P2	3 A/cm^2^	17.3	0.217
P3	6.5 A/cm^2^	12.0	0.337

**Table 4:** Test parameters.

Scanning Range of Potential (V)	Scanning Speed (mV/s)	Bath Temperature (°C)
−0.8~0.8	1	38

**Table 5:** Corrosion parameters of milling and electrochemical polishing samples.

	*E_corr_* (V/SCE)	*i_corr_* (A/cm^2^)	*β_a_* (mV)	*β_c_* (mV)	*R_p_* (Ω·cm^2^)
M1	−0.16045	2.1987 × 10^−6^	572.66	253.19	2.0113 × 10^−4^
P1	−0.13243	8.9549 × 10^−7^	961.35	251.63	9.6982 × 10^−4^
P2	0.016351	5.2474 × 10^−7^	946.04	254.26	1.6583 × 10^−5^
P3	−0.14842	1.1288 × 10^−6^	1362.2	255.14	8.2662 × 10^−4^

**Table 6:** Component values of equivalent circuit model.

	*Z*(*w*_∞_)/(Ω·cm^2^)	*Z*(*w*_0_)/(Ω·cm^2^)	*R_s_*/(Ω·cm^2^)	*R_p_*/(Ω·cm^2^)
M1	7.8015	109,700	7.8015	109,692.2
P1	9.3156	178,400	9.3156	178,390.7
P2	9.8778	237,220	9.8778	237,210.1
P3	9.5486	144,780	9.5486	144,770.5
